# Inflammation and coronary microvascular disease: relationship, mechanism and treatment

**DOI:** 10.3389/fcvm.2024.1280734

**Published:** 2024-05-21

**Authors:** Zehui Guo, Zhihua Yang, Zhihui Song, Zhenzhen Li, Yang Xiao, Yuhang Zhang, Tao Wen, Guiyun Pan, Haowei Xu, Xiaodi Sheng, Guowang Jiang, Liping Guo, Yi Wang

**Affiliations:** ^1^ Institute of Traditional Chinese Medicine, Tianjin University of Traditional Chinese Medicine, Tianjin, China; ^2^ State Key Laboratory of Component-Based Chinese Medicine, Tianjin University of Traditional Chinese Medicine, Tianjin, China; ^3^ Tianjin Academy of Traditional Chinese Medicine Affiliated Hospital, Tianjin, China; ^4^ Department of Pharmacy, The Second Affiliated Hospital of Tianjin University of Traditional Chinese Medicine, Tianjin, China

**Keywords:** coronary microvascular disease, inflammation, relationship, mechanism, treatment

## Abstract

Coronary microvascular disease (CMVD) is common in patients with cardiovascular risk factors and is linked to an elevated risk of adverse cardiovascular events. Although modern medicine has made significant strides in researching CMVD, we still lack a comprehensive understanding of its pathophysiological mechanisms due to its complex and somewhat cryptic etiology. This greatly impedes the clinical diagnosis and treatment of CMVD. The primary pathological mechanisms of CMVD are structural abnormalities and/or dysfunction of coronary microvascular endothelial cells. The development of CMVD may also involve a variety of inflammatory factors through the endothelial cell injury pathway. This paper first reviews the correlation between the inflammatory response and CMVD, then summarizes the possible mechanisms of inflammatory response in CMVD, and finally categorizes the drugs used to treat CMVD based on their effect on the inflammatory response. We hope that this paper draws attention to CMVD and provides novel ideas for potential therapeutic strategies based on the inflammatory response.

## 1 Introduction

The coronary arterial system can be conceptually divided into three compartments with progressively decreasing diameter and distinct physiology, including the epicardial coronary arteries (0.5–5.0 mm in diameter), the pre-arterioles (0.1–0.5 mm in diameter) and intramyocardial arterioles (<0.1 mm in diameter). The pre-arterioles and intramyocardial arterioles together make up the coronary microcirculation. The pre-arterioles can sense changes in coronary artery perfusion pressure and/or blood flow and regulate microcirculatory pressure by vasodilatation and contraction. The intramyocardial arterioles are the sites of myocardial metabolic exchange, and their blood flow is mainly affected by metabolites (1). CMVD is a clinical syndrome of acute and chronic myocardial ischemia caused by abnormalities in the structure and function of coronary arterioles, arterioles, and capillaries induced by atherosclerotic and non-atherosclerotic pathogenic factors (2). According to different etiologies, CMVD is classified as CMVD without obstructive coronary artery disease, CMVD with obstructive coronary artery disease, and other types of CMVD (3). CMVD is a multifactorial disease that is prevalent in cardiovascular diseases such as obstructive coronary artery disease, myocardial infarction with no obstructive coronary artery disease, ischemia with non-obstructive coronary arteries, heart failure with preserved ejection fraction, diabetic cardiomyopathy, dilated cardiomyopathy, and hypertrophic cardiomyopathy, and it can affect the pathophysiological mechanisms and prognosis of specific populations (4). Many patients with clinical symptoms of myocardial ischemia show normal or no significant stenosis on coronary angiography, and this group of patients has a higher prevalence of CMVD, as well as a significantly higher rate of mortality and adverse cardiovascular events (5). A meta-analysis (6) that included 56 studies reported a 41% prevalence of CMVD in 14,427 patients with non-obstructive coronary artery disease who met the inclusion criteria. In the setting of non-obstructive coronary artery disease, impaired CFR is present in up to 75% of patients with HFpEF and impaired CFR is a characteristic of CMVD (7, 8).

CMVD is characterised by high morbidity, low diagnostic rate and poor prognosis (9–11). Relevant studies have shown (12) that the prevalence of CMVD in the myocardial ischemia population is about 40%–64%, but only about 6.3% of the population has received timely diagnosis and treatment. Currently, assessment tools for diagnosing CMVD are divided into invasive coronary microvascular function testing methods and non-invasive coronary microvascular function examination methods. Invasive testing methods include coronary flow reserve (CFR), microvascular resistance reserve (MRR), index of microcirculatory resistance (IMR), vascular reactivity test, continuous thermodilution, and bolus thermodilution (13–18). Non-invasive testing methods include positron emission tomography (PET), cardiac magnetic resonance imaging (CMR), and transthoracic doppler echocardiography (TTDE) (1). The main drugs used in the treatment of CMVD include angiotensin-converting enzyme inhibitors, angiotensin receptor blockers, β-blockers, calcium antagonists and calcium channel blockers, etc. (19). Although there is some clinical efficacy, many patients are still hospitalized and/or undergo coronary arteriography repeatedly due to angina pectoris, which seriously affects the quality of life of patients, and at present, specific targeted drugs related to CMVD are still lacking. Therefore, it is of great clinical significance to further deepen the research on CMVD and to understand the pathogenesis of CMVD for the diagnosis and treatment of CMVD.

The pathogenesis of CMVD is complex and is associated with hemodynamic changes, oxidative stress, Ca^2+^ overload, energy metabolism, inflammatory response, platelet activation, and capillary thinning (20, 21). Inflammation is considered to be one of the key drivers of CMVD (22), which can affect the structure and function of coronary microvessels and thus lead to the development of CMVD (23–25). Basic and clinical studies have confirmed that inhibiting the inflammatory response can improve CMVD and delay its progression (26). Therefore, modulating the inflammatory response is crucial for the prevention and treatment of CMVD. In this paper, we systematically summarise the relationship between inflammatory response and CMVD, the possible mechanism of action of inflammatory response in CMVD, and the drugs for treating CMVD by inhibiting inflammatory response, with a view to providing new ideas for clinically targeted anti-inflammatory treatment of CMVD.

## 2 Relationship between inflammatory response and CMVD

### 2.1 Changes in inflammatory markers in CMVD patients

Clinical studies have found a variety of inflammatory markers to be closely associated with CMVD (27). Serum C-reactive protein (CRP), as one of the recognized inflammatory markers, is often used to evaluate the level of inflammation in the body, and elevated CRP is also closely associated with impaired vascular endothelial function in CMVD patients (28). Coronary flow reserve (CFR) is an effective indicator of coronary microcirculation and myocardial perfusion status, and it is generally accepted that in the absence of obstructive stenosis of the epicardial coronary arteries, a decrease in CFR can indirectly indicate the presence of CMVD (25). By comparing the CFR of CMVD patients with different levels of CRP to that of healthy individuals, Recio-Mayoral et al. (29). found that patients with a low level of CRP (≤3 mg/L) had a lower level of CRP (≤3 mg/L) than healthy individuals. ≤3 mg/L) in CMVD patients did not differ significantly from that of healthy individuals (*P* = 0.29), whereas the CFR of CMVD patients with high CRP levels was significantly lower than that of the group with low CRP levels (*P* = 0.005), confirming the correlation between inflammation and CMVD and the dose-dependent effect of CRP on CFR. In addition, Schroder et al. (30) found 18 biomarkers associated with CFR by analyzing biomarkers from CMVD patients, of which eight biomarkers (chemokine C-C motif ligand 16 (CCL16), chemokine CXC ligand 16 (CXCL16), peptidoglycan recognition protein (PGLYRP1), TNF receptor 1 (TNFR1), growth differentiation factor 15 (GDF15) and TNF receptor superfamily 10C (TNFRSF10C)) are associated with the pro-inflammatory pathway IL-1β/TNF-α/IL-6/CRP. In a study conducted by Suhrs et al. (25), 17 inflammatory markers were found to be negatively correlated with CFR in blood samples from CMVD patients, further confirming the strong association between inflammation and CMVD. In addition (31), a meta-analysis that included 21 studies involving 7,403 patients showed that patients with high-sensitivity C-reactive protein (hs-CRP), neutrophil-to-lymphocyte ratio (NLR), and platelet-to-lymphocyte ratio (PLR) before undergoing PCI had a significantly higher incidence of no-reflow or slow flow. Pre-coronary angiography CRP/hsCRP independently predicted no-reflow and slow flow, suggesting that high-risk patients with higher pre-procedure blood tests for inflammation-related factors can be identified in advance to prevent potential reperfusion injury as soon as possible. A study using proteomic analysis of biomarkers of cardiovascular disease also confirmed that the IL-1β/TNF-α/IL-6/CRP pro-inflammatory pathway was significantly associated with women suffering from angina pectoris combined with CMVD (30). In conclusion, the inflammatory response is closely related to the development of CMVD, and this finding suggests that advanced prediction and intervention of related inflammatory factors is important clinical guidance for the prevention and treatment of CMVD.

### 2.2 Changes in inflammatory response in animal models of CMVD

In terms of basic research, By modulating inflammation-related pathways to attenuate the inflammatory response, increase coronary microvessel density and reduce microthrombosis, CMVD progression was delayed. By establishing a MIRI mouse model, Koya found that von Willebrand factor (VWF)-mediated platelet adhesion to the microvascular endothelium aggravated the inflammatory response, resulting in impaired microvascular reflux (32). Li et al. (33) found that ICAM1, an adhesion molecule that recruits inflammatory cells from myocardial tissues, was increased in animal models of CMVD and that the expression of pro-inflammatory factors IL-6 and monocyte chemotactic protein 1 (MCP-1) was elevated in cardiac myocytes. Another study found that increased release of inflammatory factors leads to impaired vascular endothelial barrier function after coronary artery occlusion (34, 35). Qin et al. (36) found that the expression of inflammation-related factors TNF-α and IL-1β was increased in an *in vitro* and *in vivo* model of CMVD. In conclusion, inflammatory response plays an important role in the pathogenesis of CMVD.

## 3 Mechanisms of CMVD induced by inflammatory response

**Figure 1:**
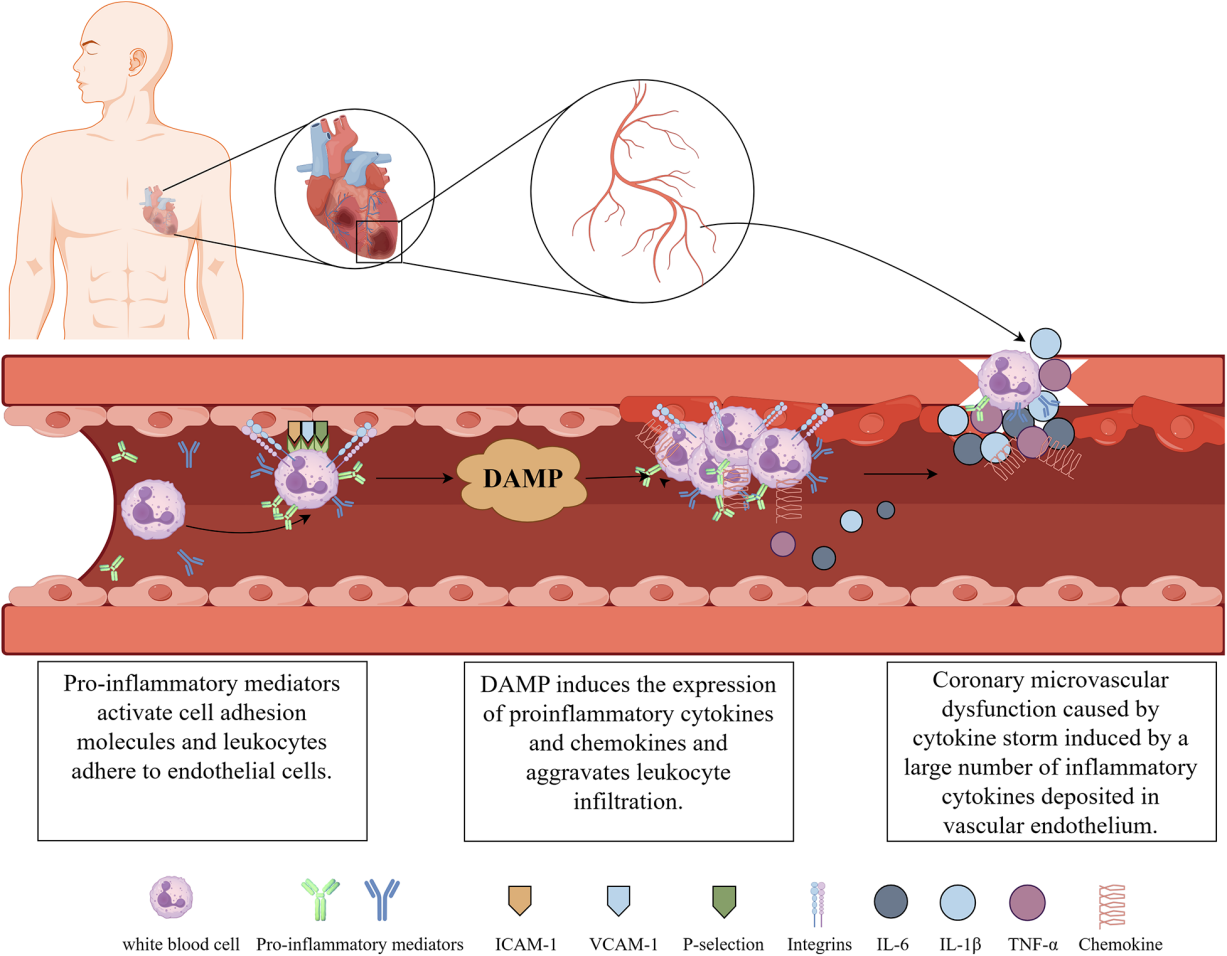
The pathogenic mechanisms for CMVD. (Illustrations by Figdraw).

The mechanisms by which the inflammatory response causes CMVD are multifaceted and interrelated. Firstly, activation of cell adhesion molecules causes inflammatory infiltration. Second, leukocyte infiltration activates the inflammatory response. In addition, inflammatory cytokines mediate CMVD development. Inflammatory response plays an important role in CMVD, and understanding and intervening in the inflammatory response is important for the prevention and treatment of CMVD (Figure 1).

### 3.1 Activation of cell adhesion molecules causes inflammatory infiltration

In CMVD, activation and adhesion of inflammatory cells to damaged tissues are key steps in inflammatory infiltration, and cell adhesion molecules play an important role in this process. Cell adhesion molecules are present on the surface of cells, and they can interact with adhesion molecules in other cells or tissues to promote cell adhesion, migration, and infiltration. Damage to cardiac tissues after CMVD generates a large number of pro-inflammatory mediators and cytokines (37, 38), which cause the activation of cell adhesion molecules, including platelet-endothelial cell adhesion molecule (P-selectin), intercellular adhesion molecule-1 (ICAM-1) and vascular adhesion molecule-1 (VCAM-1), among others. These activated adhesion molecules act as receptors and ligands, respectively, to mediate the adhesion reaction between leukocytes and endothelial cells, which becomes the initial link of inflammatory infiltration. CMVD is one of the common complications of myocardial ischemia-reperfusion injury (MIRI). Early in MIRI, the selectin family mediates the initial adhesion of leukocytes to endothelial cells, and integrins expressed by activated neutrophils (PMN) enhance the adhesion between the two, causing the initial adhesion to become a tight adhesion. PMNs are further activated and infiltrated into ischemic cardiac tissues through the endothelial cell gap induced by chemokines. Once PMN infiltrates into the tissue, inflammatory cells release inflammatory mediators (37, 38), which further exacerbate the inflammatory response and vascular endothelial damage. In dogs, ICAM-1 expression increased progressively after MIRI and was accompanied by PMN infiltration of the damaged myocardium (34). Li et al. (39) found that the expression of adhesion molecules, such as VCAM-1 and ICAM-1, could be inhibited by overexpression of histidine triplex-conjugated nucleotide binding 2 (HINT2), which enhances the endothelial barrier function of the damaged myocardium and prevents inflammatory responses. Targeted inhibition of cell adhesion factor expression reduces leukocyte-endothelial cell interactions, stimulates potent anti-inflammatory effects, and attenuates vascular inflammatory responses (40). Thus, activation of cell adhesion molecules is a key link in causing inflammatory infiltration, leading to the development of CMVD.

### 3.2 Leukocyte infiltration activates inflammatory response, causing coronary endothelial cell dysfunction

The effect of the inflammatory response on CMVD is mainly characterized by the recruitment and activation of immune cells at the site of injury (41), especially monocytes and PMN infiltration. Monocytes cross the vessel wall into the tissue after interacting with endothelial cells at the site of injury. After entering the tissue, monocytes transform into macrophages, release inflammatory mediators such as cytokines and chemokines, and activate the inflammatory response. PMN is an important component of the body's immune system. Under normal conditions, vascular endothelial cells and PMN flowing in the bloodstream repel each other to ensure microvascular perfusion. After MIRI, intracellular calcium overload and high production of oxygen free radicals lead to the degradation of cell membrane phospholipids and an increase in arachidonic acid metabolites, attracting a large number of PMN to adhere to the vascular endothelial cells and enter into the tissues. The endogenous molecules released by these cells are known as the damage-associated molecular pattern (DAMP) (42); DAMP triggers an intercellular signaling cascade response through activation of pattern recognition receptors (PRRs), leading to the expression of pro-inflammatory cytokines and chemokines, exacerbating leukocyte infiltration and endothelial cell damage, leading to coronary endothelial cell dysfunction (43). In addition, another clinical study found that elevated PMN on admission in patients with anterior wall acute myocardial infarction was associated with microvascular injury and a higher rate of long-term adverse events (44). It was observed through experimental modelling that PMN aggregation, coagulation cascade and reactive oxygen radical accumulation caused by coronary microvascular constriction and endothelial dysfunction can exacerbate disturbances in microvascular integrity (45). This further confirms that leukocyte activation is closely related to the pathogenesis of CMVD.

### 3.3 Inflammatory cytokines mediate CMVD development

Inflammatory cytokines are important mediators involved in the body's inflammatory response and the pathophysiological process of CMVD (46), and are mainly produced by vascular endothelial cells, monocyte-macrophages and vascular smooth muscle cells. Vascular endothelial cells are not only the target cells for the action of many cytokines, but also express and produce a variety of cytokines, such as TNF-α and IL-1β, upon stimulation. These cytokines affect lipid uptake and metabolism by promoting the proliferation of vascular endothelial cells and smooth muscle cells, leading to the formation of atherosclerosis and the production of large amounts of IL-6 and IL-8 involved in the stress response. The repeated proliferation of vascular endothelial cells and smooth muscle cells and the recurrent inflammatory response in this process leads to further increases in inflammatory cytokines such as TNF-α, IL-6, and IL-8, which in turn cause cellular necrosis, thrombosis, and neointima formation (47). He et al. (35) summarised that the massive production of inflammatory cytokines induces a certain degree of the cytokine storm. Inflammatory response leads to the continuous accumulation and enhancement of cytokine storm in the heart, damaged cardiomyocytes and endothelial cells release large amounts of TNF-α, which in turn promotes the increase of IL-6, and the two coordinate to promote the onset of the immune response, and the associated antibodies are produced in large quantities and deposited in the endothelium of the vessels in the form of immune complexes to form thrombi. If excessive inflammatory cytokines accumulate in the heart, it can lead to a large-scale inflammatory cascade, resulting in cardiac insufficiency and seriously affecting the prognosis (33). The inflammatory cytokine interleukin (IL)-6 is an important mediator of the inflammatory process in coronary artery disease, and may also contribute to the I/R injury in MI. Levels of IL-6 increase substantially after MI and are associated with poor short-term outcomes. In a randomized, double-blind, placebo-controlled trial, the ASSAIL-MI trial (27), patients with ST-segment elevation myocardial infarction (STEMI) who had their inflammatory response attenuated by tocilizumab, a potent agent that blocks IL-6 signaling, experienced a greater than 50% reduction in CRP levels, a lesser extent of microvascular obstruction, and a reduction in the extent of I/R injury. In summary, inflammatory cytokines play an important role in the development of CMVD due to their functions of mediating, regulating, and participating in inflammatory and immune responses. Controlling the production and action of inflammatory cytokines may become a new strategy for the treatment of CMVD.

## 4 Delaying the development of CMVD by modulating the inflammatory response

Modulation of the inflammatory response can attenuate vascular endothelial injury and delay the development of CMVD. Summarising and exploring intervention methods based on suppressing the inflammatory response may provide new strategies for the treatment of CMVD. Current methods of modulating the inflammatory response mainly include anti-atherosclerotic drugs, anti-myocardial ischemia and angina drugs and natural medicine.

### 4.1 Drug interventions

#### 4.1.1 Anti-atherosclerotic drugs

CMVD can present as diverse phenotypes in relation to atherosclerosis (AS). CMVD can occur without atherosclerosis, with non-obstructive atherosclerosis and with obstructive atherosclerosis (48). Understanding the mechanisms involved in microvascular impairment prior, during and after AS is important for risk assessment and choice of treatment. Most of the clinical CVD risk factors in CMVD patients are closely related to AS (49), e.g., the traditional risk factors for AS, smoking, hypertension, hyperlipidemia, and diabetes mellitus, may promote the development of CMVD. Primary prevention of AS by controlling risk factors may help to alleviate CMVD and angina symptoms. In obstructive atherosclerosis, plaque rupture and erosion occurs spontaneously or during percutaneous coronary intervention (PCI) after myocardial infarction. At some residual blood flow, the atherothrombotic debris is washed into the coronary microcirculation, causing physical obstruction, vasoconstriction, inflammation and microvascular dysfunction (50). Statins, antiplatelet agents, angiotensin-converting enzyme inhibitors (ACEIs) or receptor blockers (ARBs) are anti-atherosclerotic drugs (51). The anti-inflammatory effects of statins have been confirmed in experimental and clinical settings, and their inhibition of inflammatory responses not only plays a positive role in atherosclerosis, but also affects the expression of cytokines, such as TNF-α, IL-1, and IL-6, by reducing the adhesion and activation of inflammatory cells, and then repairing the endothelial damage of coronary microvessels, effectively improving myocardial ischemia and CMVD (52, 53). Aspirin is a widely used antiplatelet drug in clinical practice, and it also has anti-inflammatory effects. Aspirin reduces the expression of several inflammatory markers in cardiovascular disease (CVD), such as hs-CRP, IL-6, MCP-1, M-CSF, and TNF-α (54). In addition, aspirin inhibits the activation of NLRP3 inflammasome in a dose-dependent manner, restores endothelial barrier and permeability, and improves microvascular endothelial dysfunction (55).

#### 4.1.2 Anti-myocardial ischemia and angina drugs

Traditional antimyocardial ischemia and angina drugs, including beta-blockers, nitrates, calcium channel blockers (CCBs), nicorandil, ivabradine, ranolazine, and others. Among them, metoprolol is a commonly used β-blocker, which improves cardiac impairment in CMVD and reduces myocardial infarct size to improve microcirculation.In basic research (56), metoprolol has been found to have anti-inflammatory effects, inhibiting the expression of inflammatory cytokines such as IL-1β, IL-6 and tumor necrosis factor-α (TNF-α), inhibiting neutrophil migration and penetration, thereby alleviating MIRI (57). Diltiazem is a representative drug of CCBs, which plays a significant role in inhibiting inflammatory response in MIRI, and is often used as an active control drug in basic research on MIRI and inflammatory response (58). Nicorandil can attenuate the inflammatory response after PCI in patients with coronary heart disease (59), significantly reduce hs-CRP levels, and treat CMVD by inhibiting the expression of inflammatory factors. Ivabradine weakens the gene expression of inflammatory mediators, specifically TNFα, IL-7, IL-84β, and multiple inflammatory cell nuclei, in an animal model of ventricular remodeling. Furthermore, it provides a safeguard against ventricular remodeling and adverse cardiovascular events that arise after CMVD by limiting inflammatory responses (3).

Ranolazine is a well-described antianginal drug, its main pharmacological effects include inhibition of Na^+^, reduction of adhesion molecules, and pro-inflammatory cytokine expression, which reduces the adhesion of leukocyte activation to endothelial cells (60). In a randomized, double-blind, placebo-controlled, crossover, mechanistic trial (61), Merz et al. found that late sodium current inhibition with ranolazine may beneficially improve angina and myocardial perfusion reserve index in CMVD population with more severe CMVD.

#### 4.1.3 Natural medicine

Studies have shown that natural drugs can regulate CMVD in different ways, among which inflammation is an important intervention. Gastrodian is a natural medicine that has been used in traditional Chinese medicine for centuries to treat cardiovascular and cerebrovascular diseases, and gastrodin is its effective monomeric component. Sun et al. found that gastrodin can reduce inflammatory cell infiltration and inprove CMVD by inhibiting the NLRP3/caspase-1 signaling pathway (62). Ligustrazine, also known as Tetramethylpyrazine, is an alkaloid monomer first extracted from Chuanxiong Rhizoma (Ligusticum chuanxiong Hort.), a Chinese herb for activating circulation and removing stasis. Gao et al. found that ligustrazine exerts anti-inflammatory effects to prevent CMD via suppressing miR-34a-5p and promoting Sirt1 (63). Salvia miltiorrhiza is the most commonly used natural drug in clinical treatment of CMVD. It has significant anti-inflammatory effects. It can increase coronary blood flow, improve microcirculation and protect vascular endothelial function by inhibiting inflammation (64). Xu et al. (65) reported that the protective effect of baicalein on the heart is realized through its anti-inflammatory effect. Astragaloside IV (AS-IV) is one of the main components of the aqueous extract of Radix Astragali. Zheng et al. (66), through a meta-analysis, summarized that AS-IV alleviates the microvascular damage of MIRI by reducing NF-ĸB and TNF-α inhibiting inflammation. Many studies have confirmed that AS-IV has a significant protective effect on the heart (67–69), and the mechanism of its treatment of CMVD may be closely related to its anti-inflammatory effect.

## 5 Summary and prospect

Inflammation plays an important role in the occurrence and development of CMVD. Endothelial cell damage and inflammatory reaction interact with each other, resulting in inflammatory infiltration and injury of heart tissue. Therefore, understanding the correlation between inflammation and CMVD can provide a new direction for the prevention and treatment of CMVD. Current studies have shown that improving the treatment of CMVD by inhibiting inflammatory response may be an effective strategy. Targeted therapy for inflammatory mediators and cell adhesion molecules has shown certain efficacy. Further research and exploration is required to determine the relationship between inflammation and CMVD. Conducting more basic and clinical research related to CMVD can offer additional evidence-based support and guidance for its diagnosis and treatment. Simultaneously, an inflammation-inhibiting CMVD diagnosis and treatment system can provide the basis for personalized treatment. In conclusion, researching the relationship between CMVD and inflammation to find new therapeutic targets and develop drugs can enhance the prognosis and quality of life for affected patients.

## Glossary

ACEIs: Angiotensin-converting enzyme inhibitors
ARBs: Angiotensin receptor blockers
AS-IV: Astragaloside IV
AS: Atherosclerosis
CCBs: Calcium channel blockers
CCL16: Chemokine C-C motif ligand 16
CFR: Coronary Flow Reserve
CMR: Cardiac magnetic resonance imaging
CMVD: Coronary microvascular disease
CRP: C-reactive protein
CVD: Cardiovascular disease
CXCL16: Chemokine CXC ligand 16
DAMP: Damage-associated molecular patterns
GDF15: Growth differentiation factor 15
HFpEF: Heart failure with preserved ejection fraction
HINT2: Histidine triplex-conjugated nucleotide binding 2
hsCRP: High-sensitivity C-reactive protein
ICAM-1: Intercellular adhesion molecule-1
IL-1β: Interleukin- 1β
IL-6: Interleukin- 6
IMR: Index of microcirculatory resistance
MCP-1: Mononuclear chemotactic protein 1
M-CSF: Macrophage colony-stimulating factor
MIRI: Myocardial ischemia reperfusion injury
MRR: Microvascular resistance reserve
NF-ĸB: Nuclear factor kappa-B
NLR: Neutrophil-to-lymphocyte ratio
NLRP3: NOD-like receptor thermal protein domain associated protein 3
PCI: Percutaneous coronary intervention
PET: Positron emission tomography
PGLYRP1: Peptidoglycan recognizing protein
PLR: Platelet-to-lymphocyte ratio
PMN: Neutrophils
PRRs: Pattern recognition receptors
P-selectin: Platelet-endothelial cell adhesion molecule
STEMI: ST-segment elevation myocardial infarction
TNF-α: Tumor necrosis factor-α
TNFR1: TNF receptor 1
TNFRSF10C: TNF receptor superfamily 10C
TTDE: Transthoracic doppler echocardiography
VCAM-1: Vascular adhesion molecule-1
vWF: von Willebrand Factor
